# A Novel Test Set-Up for Direct Evaluation of Impact and Energy Absorption for Lattices

**DOI:** 10.3390/ma18173938

**Published:** 2025-08-22

**Authors:** Mohammad Reza Vaziri Sereshk, Kamil L. Kwiecien, Akib T. Lodhi, Mohammad Mahjoob

**Affiliations:** Department of Engineering, Central Connecticut State University, New Britain, CT 06050, USA

**Keywords:** Lattice, impact absorption, energy absorption, instrumented impact tester, additive manufacturing

## Abstract

The application of lattices as protective materials/structures is rapidly increasing. This requires improving impact absorption capabilities to protect goods in packaging and prevent human injuries in protective devices. This study aims to improve the accuracy of impact and energy absorption measurements for lattices, addressing the limitations of current methods such as energy-impact diagrams and instrumented drop-impact testers. A novel test setup is introduced by utilizing a modified Charpy test machine equipped with appropriate instrumentation to directly measure both energy and acceleration. Other modifications include adjustments to the machine components and the introduction of a new sandwich configuration for the test specimen, ensuring compatibility with the machine’s geometry and the test objectives. The attractiveness of the proposed test setup lies in its simplicity and efficiency. Unlike drop-impact test machines—which require complex, time-consuming, and error-prone data integration and derivation—the proposed method eliminates the need for postprocessing, as both energy and impact are recorded directly and instantaneously by the machine. The advantage over existing setups becomes particularly evident when considering that, in the presence of noise and high-frequency fluctuations—characteristic of sensor data from impact events—errors in numerical operations can range from 30% to over 100%. The functionality of the proposed test setup is evaluated through a series of experiments, and the results are compared with those obtained from existing methods. Our findings demonstrate the effectiveness of the new setup in providing accurate and direct measures of absorption parameters, offering a significant improvement over the traditional approaches.

## 1. Introduction

The energy absorption capabilities of lattice structures have been extensively studied in the literature [1]. However, their potential for impact absorption—an equally important advantage—has received relatively little attention. Protective applications, such as packaging goods, offer a clear example of where this property is valuable. Enhancing human safety is another key area, particularly in scenarios like protecting occupants of military vehicles from land mine explosions. In such cases, the blast pressure can impart significant momentum to the vehicle, resulting in life-threatening injuries, including traumatic brain injury and spinal cord compression due to rapid acceleration [2,3]. Various structural solutions have been developed for these challenges. For instance, metal-foam sandwich plates have been used for protection against air blasts [4], and blast boxes made from metallic lattices have been designed specifically to protect against land mine explosions [5]. Vaziri Sereshk and Fairson [5] also developed a crash box for civilian vehicles, designed to be mounted on the bumper to protect occupants in low-velocity collisions and to reduce vehicle damage and occupant injury in high-velocity crashes. This concept can be extended to other applications, such as protective landing structures for NASA capsules returning to Earth, or military airdrop packages impacting the ground. Another promising application involves using lattice structures as impact absorbers in helmets (constructed from polymers) and body armor (made from ceramics). Farajzadeh Khosroshahi et al. [6] explored this by testing various lattice liner configurations under different acceleration conditions to evaluate their performance in helmet applications.

Several methods exist to evaluate the impact and energy absorption characteristics of lattice structures. Gibson and Ashby [7] explored various approaches to simultaneously account for energy and impact, introducing concepts such as the Janssen factor, Cushion factor, and Rush curves. They developed the well-known energy-impact absorption diagram to identify optimal foams that offer the highest energy absorption capacity at the lowest possible stress levels—a framework primarily applied to the design of polymer foams for packaging applications. Notably, foams and lattice structures exhibit similar mechanical behavior [8]. Building on this concept, Vaziri Sereshk and Faierson [5] extended its application to metallic lattices and presented energy-impact diagrams for various lattice topologies. They further employed this approach to propose a flexible design methodology for parametric gradient lattices, enabling control over both impact response and energy absorption levels [9]. However, this remains a hybrid approach—combining experimental and analytical methods—intended primarily for design purposes. There is still a need for dedicated test setups to directly measure the impact and energy absorption performance of actual lattice samples. Moreover, it is important to note that energy–impact diagram is based on quasi-static compression tests, which do not accurately simulate high strain-rate conditions encountered in many real-world impact scenarios.

Several impact testing machines and methods have been developed to evaluate the performance of lattice structures under dynamic loading. Varna et al. [10] fabricated an instrumented drop-weight impact tester equipped with strain gauges and an accelerometer to measure impact force and acceleration. Similarly, a load cell and accelerometer [11] or speed sensor in combination with a load cell [12] were used as multi-sensor system in other studies. However, Al Rifaie et al. [13] used only a single accelerometer, then post-processed the recorded acceleration data to derive velocity, displacement, and force histories. Force was calculated using Newton’s second law of motion. It is important to note that in such drop-weight impact tests—where the penetration of the impactor head into the specimen is recorded—the absorbed energy can, in theory, be determined by calculating the initial gravitational potential energy of the falling hammer [14], assuming the hammer comes to a complete stop after impact. However, reaching the full energy absorption capacity of a lattice requires the specimen to be fully densified, which complicates the determination of corresponding potential energy. In practice, the hammer may or may not come to a full stop and could rebound, making it difficult to accurately quantify the absorbed energy. Although some machines include rebound-catching mechanisms to halt the impactor during its second descent, the overall kinematics of the impact event remains complex. As a result, multiple instruments are often required, and substantial post-processing of the data is necessary to calculate the absorbed energy—typically by integrating the area under the recorded load–displacement curve. Consequently, the energy absorption is not measured directly by the machine but must be derived from processed experimental data. These numerical processes often introduce a considerable amount of error, which can significantly compromise the accuracy and reliability of the measured data. For data with high-frequency fluctuations and noise—such as sensor data recorded during impact events—this error can range from 30% to over 100% [15].

In contrast, the Charpy test machine directly measures the impact energy. It uses a swinging pendulum to strike and break a notched specimen. The difference in the pendulum’s height before and after impact represents the energy absorbed by the specimen during fracture. In this study, a Charpy test machine is equipped with appropriate instrumentation to measure the impact as well. A new test specimen is fabricated as a sandwich with components carefully designed according to the space available as the specimen support and functionality requirements. It is designed to have lattice core (middle of specimen) densified fully before the fracture of the specimen and the absorbed energy can be read directly from the graduated display of the machine. The aim of the present study is to investigate the feasibility and functionality of the proposed test set-up. This fills the gap in literature as the need for concurrent direct measurement of energy and impact absorptions for lattices is addressed. Indirect measurement and postprocessing on the recorded data in the presence of high-frequency fluctuations make any differentiation and integration on the data challenging and the result of postprocessing can be unreliable.

## 2. Theory/Calculation

### 2.1. High-Strain-Rate Testing Methods

A comparative overview of various impact and high-strain-rate testing methods commonly used in materials science and mechanical testing is presented in Table 1, including Drop Weight, Split-Hopkinson Pressure Bar (SHPB), and Charpy tests [16,17,18]. Each method has its own strengths and limitations, depending on the material type, strain rate range, and test objectives.

### 2.2. Calculation of Absorbed Energy

**Figure 1:**
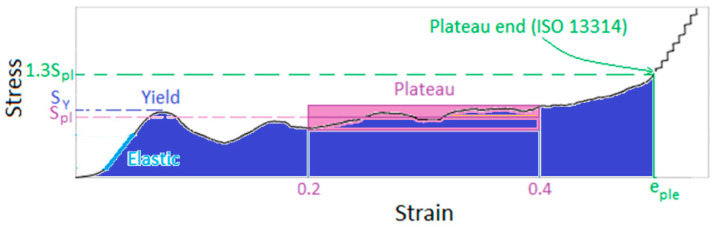
Schematic indicating terms and definitions.

Based on ISO 13314 [19] for compression test, compressive stress is defined as the ratio of compressive force and initial cross-sectional area. Compressive strain is the ratio of overall compressive displacement and initial length. Plateau stress, *S_pl_* is defined as the mean stress between the stress values at 20% and 40% (or 30%) compressive strains. Plateau end, *e_ple_* corresponds to the point on the representative diagram at which the stress is 1.3 times the plateau stress. Energy absorbed, *W*, is calculated using Equation (1).

(1) $$W=\frac{1}{100}\int_{0}^{e_{0}}\sigma de$$

where *W* is the energy absorption per unit volume (MJ/m^3^) and *σ* is the compressive stress (N/mm^2^). *e* is overall compressive displacement divided by the initial height (gauge length) of the test specimen and $e_{0}$ is upper limit of the compressive strain (%). In this study, this energy is calculated using trapezoidal rule for the area under the strain-stress curve up to the plateau end with the strain value, *e_ple_*. This is shown with indigo color in schematic diagram in Figure 1. *W_e_* is the volumetric energy absorption defined by the energy absorbed per unit volume of the lattice.

### 2.3. Calculation of Impact

Selecting an appropriate parameter to represent impact in protective applications remains a subject of debate. In buried-blast protection studies, it is common to correlate the blast impulse with the resulting deformation or damage to the structure [20,21,22,23]. Yet, impulse is not generally used as a criterion for human safety. Instead, the AC 21-22 standard (Injury Criteria for Human Exposure to Impact) [24] sets survivability thresholds based on the acceleration and force experienced by occupants in civil aircraft. Similarly, Gibson and Ashby [7] propose using peak force as the key impact parameter, recommending that it remains below a certain threshold to prevent injury or structural damage. This approach has been applied in the design of metallic lattice structures, where the maximum crushing force serves as a representative impact metric [5]. In other applications, structural acceleration in response to impact is considered as the parameter to represent impact. This was used to develop sandwich panels for air-blast protection [4] and helmet lattice liners to protect athletics [6]. Therefore, both peak force and acceleration have been recognized as meaningful and practical indicators of impact in the design and evaluation of protective devices and systems. In this study, peak force represents impact for energy-impact diagram developed based on compression test data, while recorded acceleration represents impact for novel pendulum impact testers.

### 2.4. Development of Energy-Impact Diagram

Energy-impact absorption diagram shows the normalized cumulative energy absorption per unit volume plotted against the normalized stress [7], while the normalized stress represents the impact. Cumulative energy absorption per unit volume (*W*) and Plateau stress (*S_p_*) are normalized by elastic modulus of solid material (*E_s_*). The Shoulder point on the diagram is defined as the optimum point for design. This is the point where a significant change in the slope is observed on the energy-impact curve. Deformation beyond this limit leads to a sharp rise for the load and impact, which implies transferring significant load to the object.

**Figure 2:**
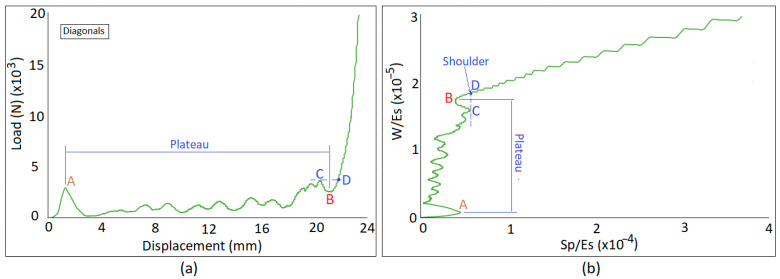
Correlation of load–displacement diagram (**a**) and energy-absorption diagram (**b**).

As an example, Figure 2 correlates the load–displacement diagram recorded by the compression machine and the energy-impact diagram calculated for a lattice sample. The ends of the Plateau region are indicated by A and B on the diagrams in Figure 2a,b and based on the definition presented in Ref. [7], point B would be the Shoulder. However, to be able to fully densify all lattice cells and benefit from the entire Plateau, a larger load than point B is needed to pass the points A and C. Therefore, instead of point B in Figure 2a, point D is considered as the Shoulder. This is obtained by intersecting the corresponding diagram by a vertical line passing through the rightmost point on the Plateau region (point C in Figure 2).

## 3. Material and Methods

Equipment and manufacturability are the limiting factors for material selection. The energy capacity of Tinius-Olsen (Horsham, PA, USA) Charpy machine used in this study is limited to 358 J. Therefore, lattice specimens should not be made from very high strength polymers. Stereolithography (SLA) is the right technology capable of 3D-printing delicate features for lattices. However, depending on the equipment used and the resin, there are thresholds for geometry and size that should be followed to obtain successful prints without defects. Minimum dimensions were considered to design lattice samples.

### 3.1. Selection of Material

**Figure 3:**
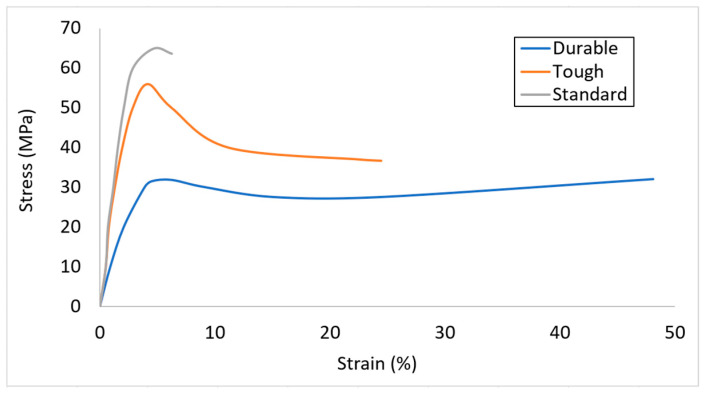
Characteristic diagrams from tensile test [25].

Finding the right polymer for this application requires a balance between multiple properties including tensile and impact strength (toughness), flexural and tensile moduli and elongation at fracture/rupture. The Formlabs Engineering (Somerville, MA, USA) resin was used for 3D-printing of the lattice samples by SLA technology [25]. Three choices were considered including Standard, Tough, and Durable resins. Standard resin is introduced for general-purpose prototyping. Tough and Durable resins are comparable to polypropylene (PP) and polyethylene (PE), respectively. The stress–strain diagrams taken from the tensile tests of the samples printed from these three resins are shown in Figure 3 [25]. Table 2 summarizes the mechanical properties [25].

Durable resin has the lowest tensile strength, the highest impact strength and the largest elongation at break, compared to the other SLA materials. These are the important properties required for protective applications. Therefore, this resin was used in this study.

### 3.2. Sample Preparation

Face Centered Cubic (FCC) topology was considered for cells. The following manufacturability thresholds were considered to optimize the lattice design and reduce failures of Formlabs resins used in printing by Form 2 printer [26]:

- Maximum unsupported overhang length is 3.0 mm.
- Minimum unsupported overhang angle is 19° from level.
- Minimum vertical-wire diameter for a 7 mm-tall wire is 0.4 mm and for a 30 mm-tall wire is 1.5 mm

**Figure 4:**
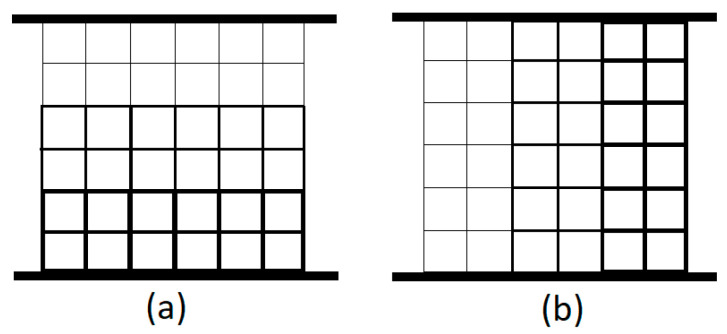
Illustrative schematics for gradient samples; (**a**) series arrangement, (**b**) parallel arrangement.

To evaluate the proposed test setup across a variety of scenarios, two known step-gradient designs were used in addition to uniform lattice core. The diameter of the struts was adjusted in three stepwise increments across the structure: perpendicular to the loading direction in the parallel configuration, and along the loading direction in the series configuration. The gradient configurations are illustrated schematically in Figure 4.

The authors were interested in comparing the effectiveness of different gradient approaches. To ensure a fair comparison, all three gradient samples were fabricated with identical weight and overall cubic volume, maintaining the same volume fraction (VF). Their performance was then evaluated against equivalent uniform (non-gradient) samples, representing traditional design methods. Each sample comprised six unit cells in each direction, as recommended in the literature [27]. The length of the strut for all samples was 5 mm. Table 3 summarizes the strut diameters for gradient and uniform samples.

**Figure 5:**
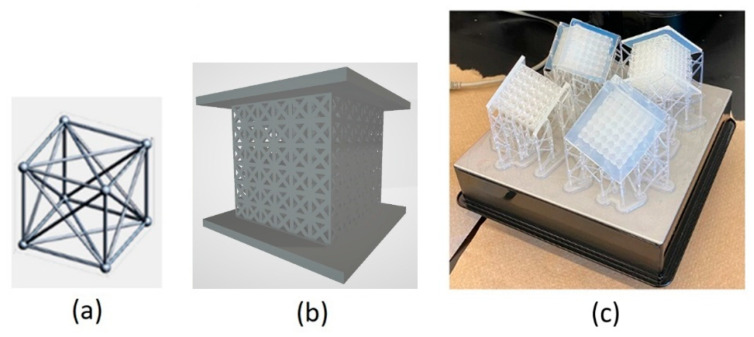
Lattice sample; (**a**) FCC unit cell geometry, (**b**) CAD model of lattice sample, (**c**) printed sample orientated on build-plate.

Figure 5a shows the geometry for the unit cell. The threshold of 0.4 mm for strut size was considered based on the available recommendation [26]. To achieve a homogeneous structure, the samples were oriented at 45° around two axes and printed on one corner (Figure 5b).

### 3.3. Sensors and Instrumentation

Instrumentation plays a central role in the design and development of a new test setup. Accelerometer is used to measure change in acceleration of hammer after strike in pendulum impact tester. This reflects the impact absorption behavior of the lattice specimen. Certain fundamental requirements must be considered when designing a system to measure and process transient signals, particularly shock or impulse events. In this study, a hammer strikes an object which generates a sudden transient excitation (shock pulse) on the specimen/structure. A modern data acquisition system consists of a measurement chain that includes a sensor—typically with an analog output—an appropriate signal conditioning unit (such as a charge amplifier or filter), followed by electronics for sampling and analog-to-digital (A/D) conversion, and finally, computer interfacing. For this application, the components of the measurement chain must be carefully selected based on the characteristics of the input signal (in this case, a shock pulse) and the specific information required from the digital output, which is ultimately stored as a dataset (vector or array) on the computer.

The common sensor used to measure acceleration is an accelerometer. Piezoelectric type of accelerometer is suitable choice to measure a highly dynamic/transient signal (shock) [28]. The ICP/IEPE piezoelectric accelerometer used here transforms the input acceleration to charge (current) which is then converted to voltage by the internal electronics integrated in the sensor. These accelerometers offer a wide measurement frequency range (from low frequencies about 1 Hz to high frequencies around 50 kHz) and are available in a range of sensitivities, weights, sizes, and shapes [28]. The frequency range and the number of axes of measurement are determined by the test requirement. For this application, parameter to measure is a typical low-level transient shock (<500 g) applied along a single-axis (direction of the impact) for a duration of few milliseconds. Therefore, the ICP^®^ Model 352C23 from PCB Piezotronics (Buffalo, NY, USA) [29] was selected as the acceleration sensor. Table 4 summarizes the specifications of the sensor [29].

The sensor is connected via a low-noise cable to a digital USB signal conditioner (PCB 485B39, PCB Piezotronics, Buffalo, NY, USA), which serves as the core component of the data acquisition (DAQ) system. This module digitizes the sensor’s analog output voltage and transmits the corresponding data to the computer via a USB interface [30]. The 2-channel, USB-powered, ICP^®^ signal conditioner (PCB Piezotronics, Buffalo, NY, USA) with USB digital output features a 24-bit A/D convertor with a frequency range of 0.8 Hz to 20,700 Hz that offers sufficient resolution and sampling rate to satisfy the requirements for the desired measurement of the transient impact signal. The data transferred via the USB port can be read and analyzed by appropriate software. SpectraPLUS-SC software (Sequim, WA, USA) [31] was used to read the acceleration data and do the rest of processing steps and analyses (Section 4.2.2).

## 4. Results and Discussion

Prior to testing by the proposed setup, it is important to evaluate the samples’ energy absorption and impact characteristics through a validated design methodology. The energy-impact diagrams (as described in Section 2.4) are generated to determine absorption properties.

### 4.1. Energy-Impact Diagrams

**Figure 6:**
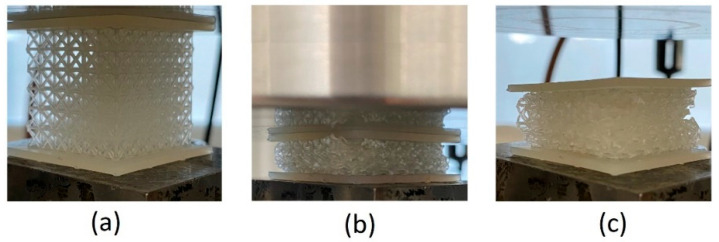
Deformation of lattice sample; (**a**) at the beginning of test, (**b**) at the end of test, (**c**) right after lifting the platen.

An Instron (Norwood, MA, USA) 5585H universal testing machine was used for compression testing. The compression tests were conducted on the FCC lattice samples described in Section 3.2. The load and displacement data were recorded in each test. The speed of test was 1.8 mm/min to reach a strain rate of 10^−3^ s^−1^ and was maintained during the test. Figure 6 shows snapshots of the deformation for the lattice sample at the beginning and end of test as well as the final configuration of the sample after lifting the compression platen.

**Figure 7:**
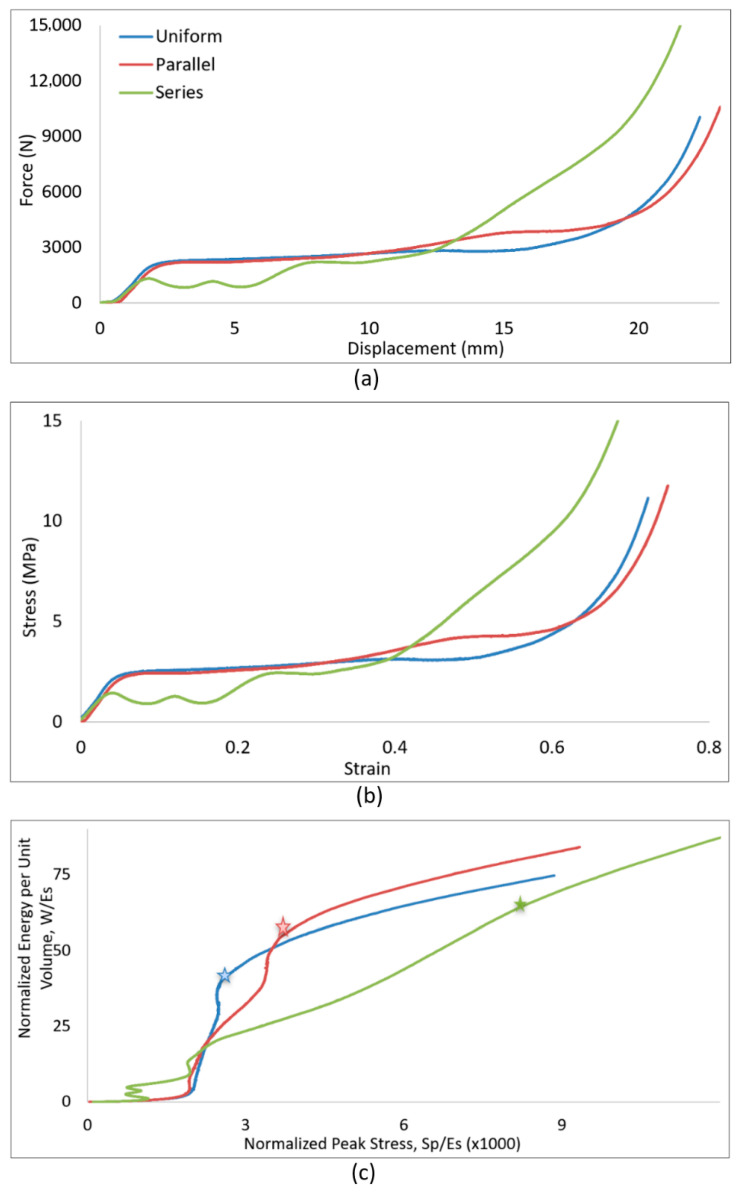
Design diagrams for FCC samples; (**a**) Recorded load–displacement data for compression test, (**b**) Characteristic diagram, (**c**) Energy-impact diagrams.

Figure 7a shows the recorded load and the displacement data for FCC samples. Based on ISO 13314 [19], the compressive stress and strain were calculated using the load and displacement data recorded by the test machine and the sample size (the procedure was explained in Section 2.2). Figure 7b shows the calculated stress–strain data. The computation described in Section 2.4 was conducted on the data taken from the compression test, and the following energy-impact diagrams (shown in Figure 7c) were obtained for the FCC samples. The ‘asterisk’ marks on the curves indicate the corresponding Shoulder points.

From a design standpoint, the location of the shoulders in Figure 7c indicates that both parallel and series gradient lattices exhibit higher energy absorption compared to the uniform lattice. However, this improvement comes at the cost of increased impact forces, with the series arrangement generating the highest impact. This behavior is further investigated using the new test setup in the next phase, which focuses on practical implementation—where performance may deviate from the optimal conditions assumed during the design stage.

### 4.2. Direct Measurement of Energy and Impact

#### 4.2.1. Test Set-Up

**Figure 8:**
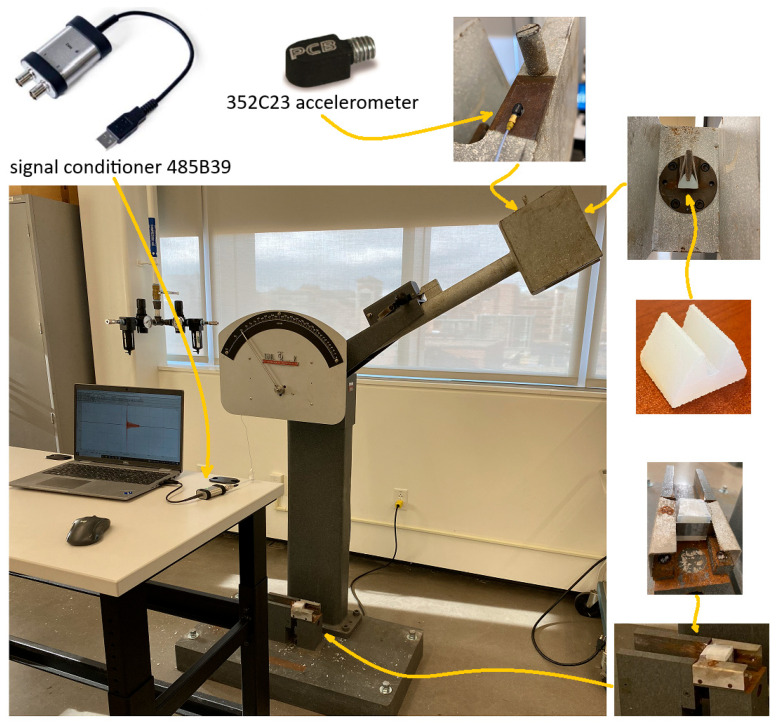
Instrumented Charpy test machine; accelerometer/sensor, striker cape, and test sample.

A Charpy impact test is generally used to measure the energy needed to break a notched sample, where the absorbed energy of the impact is directly shown on the graded display of the machine. A novel approach is presented here to equip this machine with proper instruments and measure both the impact and energy concurrently. Acceleration is the main parameter evaluated as the representative of impact. As explained in Section 3.3, an accelerometer (ICP^®^ Model 352C23) was selected as the suitable sensor to measure the acceleration [29]. Table 4 summarizes the sensor specifications. The sensor is installed on the pendulum rod at the hammer section, placed parallel to the striker head. Figure 8 shows the location and proper orientation of the sensor on the hammer. If regular striker with sharp edge hits the lattice sample, it penetrates the specimen and splits it into two halves. To distribute the impact load evenly on the faceplate of lattice core, a new striker cape (Figure 8) was designed to cover the striker edge. The flat face of the striker cape was inclined with a small angle to make the striking face parallel to the faceplate of the specimen at the onset of strike. It was 3D-printed from Durable resin in this study. In the case which the striker cape breaks during a test, the test should be repeated with a new cape. Fabricating the striker cape from metal prevents this issue.

**Figure 9:**
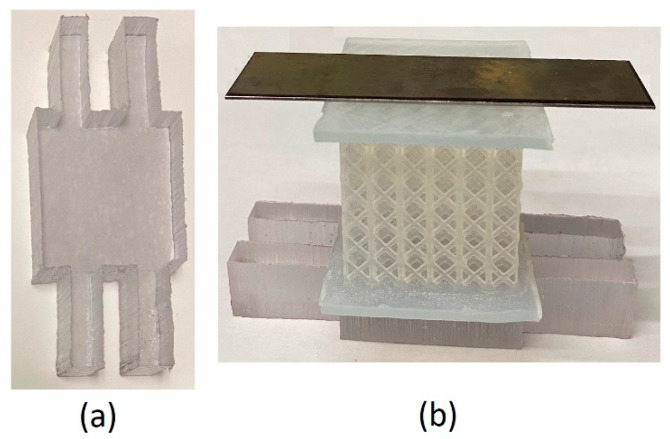
Test sample; (**a**) back-plate, (**b**) components of the sandwich sample including front-plate (**top**), lattice core (**middle**), back-plate (**bottom**).

The test sample was designed as a sandwich including the front- and back-plates and the lattice core. The back-plate was added as the support for back of the lattice core while it is compressed by the striker cape face. As shown in Figure 8, the back-plate leaning on the anvils should be strong enough to hold the right face of the sample and have its core fully densified before breakage. This is essential to reach the capacity of lattice core for absorption of energy and impact. Different materials and geometries were examined and after many modifications, design was finalized to the geometry indicated in Figure 9a. Back-plate is made from polycarbonate plastic with a thickness of 12.2 mm. Its geometry was selected to cover the width and height of the existing slot for specimen support. This eliminates the possibility of tilting during compression. The middle square portion distributes the pressure evenly on the back of the lattice core. The front-plate guides sample along the axis of support canal during the test. It is made from thin steel sheets to prevent its breakage against hammer strikes. Three parts of the sandwich specimen (Figure 9b) were bonded by a small drop of Supper Glue and held together tightly by tape.

#### 4.2.2. Data Collection and Analysis

**Figure 10:**
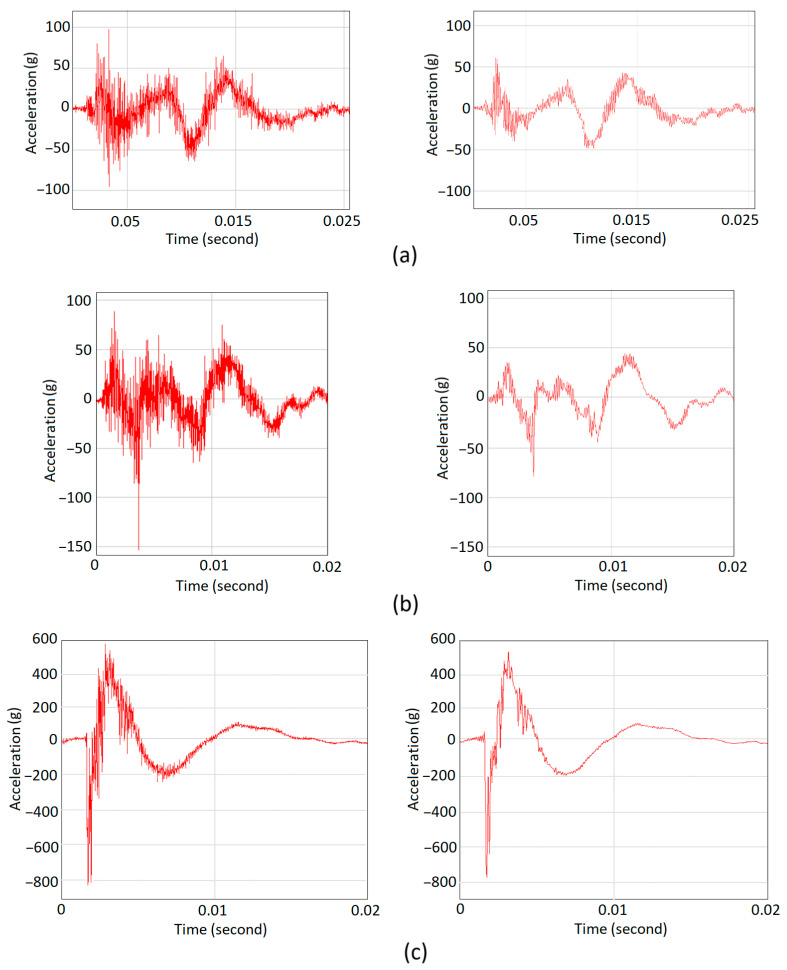
Recorded acceleration, original (**left**) and smoothed (**right**); (**a**) Parallel gradient FCC, (**b**) Uniform FCC, (**c**) Series gradient FCC.

Each FCC specimen was placed on the support and the Charpy test was performed by releasing the pendulum to strike the specimen. SpectraPLUS-SC software [31] was used to record the output signal from the accelerometer. It was already converted to digital values and transmitted via the serial USB interface to the computer. The diagrams on the left side of Figure 10 demonstrate the variation of the measured acceleration of the hammer during the strike. To smooth acceleration history, high-frequency oscillations corresponding to the frequencies beyond 7800 Hz were filtered out. This threshold was chosen after a few trials to eliminate the background noise and the higher frequencies which are associated with the machine’s rigid structure. A similar procedure is recommended by ASTM D7136M-20 [32] to smooth time history. The smoothed diagrams were placed next to the original ones on the right side of Figure 10. The absorbed energies were read out directly on the graded display of machine. Table 5 summarizes the results for energy and maximum acceleration. “g” refers to the gravitational constant.

**Figure 11:**
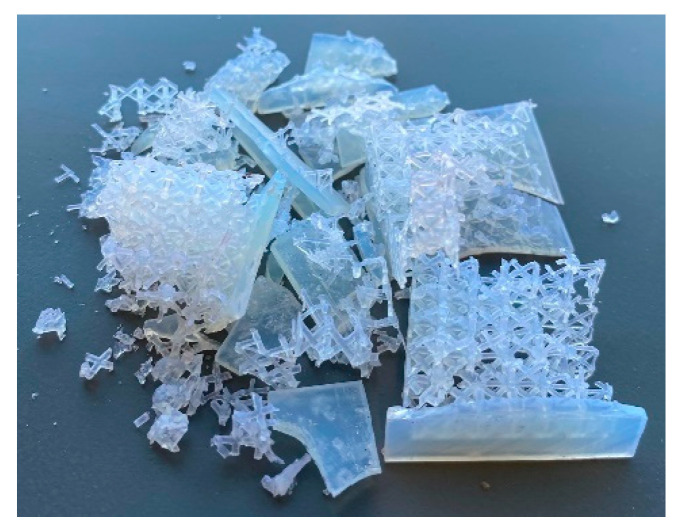
Shattered specimen after Charpy test.

As shown in Table 5, the maximum acceleration values changed after smoothing; however, the relative performance ranking remained unchanged. As anticipated from the design analysis using the energy–impact diagram (Section 4.1), the series arrangement exhibits the highest impact among all specimens, while offering only a slight improvement in energy absorption. In contrast, the parallel gradient lattice absorbs less energy but does so at a lower impact level compared to the uniform lattice. This outcome contradicts the original design objectives, which targeted optimal performance at the Shoulder point on the energy-impact diagram. It should be noted that the Shoulder point on the energy-impact diagram is considered as the optimum point for design purposes including determining thickness of lattice core. Energy and impact of Shoulder are desired values, not the actual values. However, the proposed test method measures these values. These results highlight that optimal performance predicted in the design phase is not always achievable in practice. Another difference is the sample condition after the test. Shoulder is associated with full densification of the lattice core, while proposed test set-up measures the energy and impact needed to shatter the lattice core. Figure 11 shows an example of the shattered specimen. The difference becomes evident when compared with the densified sample under compression in Figure 6c, which is still intact.

It should be noted that the result of the modified Charpy test cannot be compared with the result of discussion on energy-impact diagrams in Section 4.1. The first reason is the speed of the tests. Compression tests were conducted under quasi-static conditions, while the Charpy test is categorized as a low-velocity impact test. In addition, acceleration is the main parameter that represents the impact in the proposed approach, while force/stress is the representative parameter in the energy-impact method. Direct comparison of these two parameters is not possible.

It should be considered that the sandwich specimen consists of the front- and back-plates as well as the lattice core. Therefore, the data presented in Table 5 cannot be used as the energy and impact properties of individual lattices. Nevertheless, this test method can be employed to compare the performance of different lattice designs for a specific protective application.

## 5. Conclusions

The energy-impact diagram summarizes the data for the design of plastic foam used in protective applications. While the application has been extended to lattices, it is limited to design purposes including calculation of dimensions for a lattice core. The experimental observations indicate that the targeted optimal performance may not be fully achievable in practice. Therefore, the developed analytical approach cannot be employed to determine the properties of an existing lattice for high-strain-rate crashing applications. In this study, a Charpy test machine was equipped with proper instruments to measure the impact and energy directly. Several trials were conducted to determine geometry and material for the components of the sandwich test specimen. The acceleration was measured, and the energy was read from the graded display on the Charpy test machine. The most important feature of the proposed test setup is its ability to provide direct measurements, thereby eliminating the significant errors typically associated with numerical post-processing of high-frequency fluctuating data. The acceleration data were smoothed by removing high-frequency oscillations associated with background noise and the rigid structure of the testing machine. This smoothing procedure does not compromise the accuracy of the measurements and results in a clearer dataset for analysis. This test set-up proved to be suitable to evaluate the superiority for impact and energy absorptions of different lattice designs. The test set-up was developed to reach the shattering stage after striking the lattice core. Therefore, the result demonstrates the ultimate limits suitable for protection applications.

Further studies should be conducted to evaluate the performance of lattices with different topologies, densities, strut geometries, and filler materials. Considering the proposed composite configuration for the specimen, further calculations may exclude the contribution of the back plate to energy absorption and impact response, isolating the intrinsic properties of the lattice structure.

## Figures and Tables

**Table 1:** Comparison of high-strain-rate and impact testing methods [16,17,18].

Method	Purpose	Strain Rate Range	Sample Size	Data Output	Advantages	Limitations
**Charpy Impact Test**	Measures material toughness (energy absorption in fracture)	~10^2^ s^−1^ (low strain rate)	Standardized notched specimens (ISO/ASTM)	Impact energy, fracture appearance	Simple, fast, low cost, standardized	Limited to brittle/ductile transition; not true stress–strain; low strain rate
**Drop Weight Test**	Fracture behavior, toughness, or energy absorption under impact	~10^2^–10^3^ s^−1^	Varies (larger than Charpy)	Force, energy absorbed	Customizable loading rate; useful for large/complex specimens	Less standardized; can be noisy; strain data limited
**Split-Hopkinson Pressure Bar (SHPB)**	High-strain-rate stress–strain behavior	~10^3^–10^4^ s^−1^	Small cylindrical/dogbone samples	True stress–strain, strain rate, modulus	Accurate strain rate control; dynamic behavior	Complex setup; requires strain gauges, assumptions (1D wave propagation)
**Taylor Impact Test**	Study dynamic deformation & failure; validate simulations	~10^4^ s^−1^	Cylindrical rods	Deformed profile, high-speed imaging, sometimes strain	Visualizes failure modes; simple in concept	Limited quantification; post-mortem analysis
**Izod Impact Test**	Similar to Charpy, for plastics/metals	~10^2^ s^−1^	Notched specimens	Energy absorbed	Easy, standardized for plastics	Similar limitations to Charpy
**Gas Gun/Projectile Impact**	Penetration, ballistic performance, high-energy impacts	~10^4^–10^6^ s^−1^	Varies, often large or armor samples	High-speed imaging, strain fields, damage	Simulates real impacts; extreme conditions	Expensive, complex setup, safety concerns

**Table 2:** Properties of the products made from different resins [25].

Property	Standard	Tough	Durable
IZOD impact strength (J/m)	25	38	109
Elongation at break (%)	6.2	24	49
Tensile strength (MPa)	65.0	55.7	31.8
Tensile modulus (GPa)	2.80	2.80	1.26
Flexural modulus (GPa)	2.2	1.6	0.82

**Table 3:** Size and VF for density gradient lattice samples.

Topology	Strut Diameter (mm)		Volume Fraction
	Gradient	Uniform	
FCC	0.8, 1, 1.3	1.04	0.277

**Table 4:** Specification notes for a 352C23 accelerometer [29].

Sensitivity (±20%)	5 mV/g
Measurement Range	±1000 g pk
Frequency Range (±5%)	2.0 to 10,000 Hz
Frequency Range (±10%)	1.5 to 15,000 Hz
Frequency Range (±3 dB)	0.7 to 25,000 Hz
Resonant Frequency	≥70 kHz
Broadband Resolution (1)	0.003 g rms
Non-Linearity	≤1%
Transverse Sensitivity	≤5%

**Table 5:** Measured absorbed energy and maximum acceleration.

Sample	Absorbed Energy (J)	Maximum Acceleration (Original)	Maximum Acceleration (Filtered)
FCC-Parallel	291	−95 g	−50 g
FCC-Uniform	341	−155 g	−75 g
FCC-Series	347	−820 g	−770 g

## Data Availability

The original contributions presented in this study are included in the article. Further inquiries can be directed to the corresponding author.
